# “Brain not right” and “lonely in a crowd”: unveiling the central architecture of psychopathology in obsessive-compulsive disorder

**DOI:** 10.3389/fpsyt.2026.1823876

**Published:** 2026-05-18

**Authors:** Lianlian Xu, Yue Wu, Lina Zhang, Huanzhong Liu, Shuangyue Zhu

**Affiliations:** 1 Anhui Medical University, Hefei, China; 2 Affiliated Mental Health Center & Hangzhou Seventh People’s Hospital, Zhejiang University School of Medicine, Hangzhou, China; 3 Chaohu Hospital of Anhui Medical University, Hefei, China

**Keywords:** loneliness, metacognition, network analysis, obsessive-compulsive disorder, obsessive-schizophrenic spectrum

## Abstract

**Background:**

The co-occurrence of obsessive-compulsive disorder (OCD) and psychotic-like symptoms suggests the existence of the “obsessive-schizophrenic spectrum”, and this study uses network analysis to explore their interaction mechanism.

**Methods:**

A total of 528 patients with obsessive-compulsive disorder (OCD) were included. A symptom network was constructed based on the Yale-Brown ObsessiveCompulsive Scale (Y-BOCS), Symptom Checklist-90 (SCL-90), Self-Rating Anxiety Scale (SAS), and Self-Rating Depression Scale (SDS). Core nodes were identified through centrality indicators.

**Results:**

“Brain not right” (SCL90) has the highest betweenness centrality and serves as a crucial metacognitive mediator connecting symptom clusters. Lonely in crowd (SCL77) has the highest strength, maintaining network activation. unusual thoughts (SCL68) and Somatic concern (SCL87) emerged as a bridge node linking obsessivecompulsive symptoms to subjective experiential and thought-perceptual disturbances associated with the psychosis spectrum. Malignant loops such as “obsessive interference – collapse of metacognitive evaluation” have been identified.

**Conclusions:**

The catastrophe of brain functions and the sense of loneliness are the central hubs which drive the complexity of OCD symptoms. In addition to traditional treatments, correcting metacognitive beliefs and improving social isolation are crucial for preventing the deterioration of disease.

## 1 Introduction

Obsessive-compulsive disorder (OCD) is a mental disorder characterized by selfinconsistent intrusive thoughts (obsession) and mental or physical compulsive behaviors (compulsions), often resulting in significant distress and functional impairment (1). Although OCD was previously classified as an anxiety disorder, clinical and neurobiological evidence shows that it has a high degree of heterogeneity (2). This heterogeneity is extremely remarkable, and two confirmed patients even may exhibit completely distinct and non-overlapping symptom patterns (3). Besides the core symptoms, some patients with OCD also show significant “psychotic experiences”. Research indicates that there is a marked association between OCD, schizotypal traits and psychotic symptoms, as all involve unconventional or bizarre thinking patterns (4). Compared to the general population, patients with OCD have a higher proportion of psychotic-like symptoms such as hallucinations, delusions or thought disorders (5, 6). A substantial overlap between paranoid ideation, hallucination proneness, OCD symptoms and metacognitive beliefs has been confirmed by further study in non-clinical samples (7). Clinical patients with these psychotic features tend to have an earlier age of onset, more severe symptoms and a poorer prognosis (8).

Research on the pathological mechanism of OCD often has limitations when dealing with psychotic symptoms. Early viewpoints tended to regard the accompanying delusions as transient reactive affective disorders or paranoid psychoses, rather than equivalent to schizophrenia (9). Although most patients are aware of the irrationality of their obsessive-compulsive behaviors, those with a high fusion of obsessions and behaviors may exhibit poor insight, sometimes reaching delusional levels. According to statistics, approximately 21% to 36% of patients with OCD have poor insight and about 4% present with delusional symptom (8). However, this macroscopic perspective based solely on diagnostic classification overshadows the importance of the microscopic interaction mechanisms among symptoms. The Inference-Based Approach (IBA) posits that all intrusive thoughts are essentially a form of reasoning process. During this process, the certainty of sensory information is substituted with doubts arising from inferential confusion, leading patients to be convinced that a certain specific possibility is reality (10). While theoretically acknowledged, we still lack empirical evidence to systematically explore at the micro-network level how the psychotic and paranoid factors act as key hubs that maintain and drive the complex symptom co-occurrence loops among OCD, anxiety and depressive symptoms.

Network analysis provides a new theoretical framework to overcome this limitation. This theory posits that mental disorders don’t stem from a single underlying cause but are instead constituted by a complex network system of direct causal interactions between symptoms. When connectivity between symptoms is sufficiently tight, a self-sustaining feedback loop will form, driving the network into a hysteresis condition. In this condition, symptoms continue even after the initial triggering event is removed, thereby contributing to the development of mental health disorders (11). Under this framework, nodes exhibiting high centrality are regarded as key drivers in maintaining the state of disease, while the connections linking distinct symptom clusters serve as bridges for transdiagnostic symptom co-occurrence. Although network analysis has been extensively applied to the study of symptom co-occurrencebetween OCD, anxiety and depression (12). limited studies have incorporated specific psychotic and paranoid items from the SCL-90 into such models. Thus, the structural role of the psychotic dimension in OCD remains poorly understood.

This study aims to fill the current gap by analyzing clinical data from the 528 adolescent outpatients aged 14 years or older with OCD. A comprehensive symptom network was developed, integrating the core OCD symptoms (Y-BOCS), emotional symptoms (SAS/SDS), and specific psychotic and paranoid factors from the SCL-90. Within the symptom network, we aimed to identify which symptoms play a crucial role in maintaining network stability and to pinpoint key bridge pathways, thereby revealing potential microscopic interaction mechanisms.

## 2 Methods

### 2.1 Participants and procedures

This retrospective study analyzed a cohort of 528 individuals, aged 14 years and older, who were clinically identified with obsessive-compulsive disorder (OCD) between 2022 and 2024 at least. To be eligible for inclusion, participants were required to submit a baseline demographic survey and complete a comprehensive battery of psychometric evaluations, comprising the Y-BOCS, SAS, SDS and SCL-90.

Furthermore, participants also were required to have a confirmed clinical diagnosis of OCD based on a structured interview aligned with ICD-10 diagnostic standards. The study was conducted in accordance with the Declaration of Helsinki and received approval from an institutional ethics committee (approval number: [2026-009-01]).

### 2.2 Data cleaning

The datasets for the Y-BOCS, SAS and SDS scales contained partial missing values in the current study. The evaluation of missing data patterns indicated an overall missing rate of 5.85%. Little’s MCAR test supported the assumption that data was Missing Completely at Random (MCAR) (*p* > 0.05). Accordingly, we employed the *mice* package in R to perform Multiple Imputation using the Predictive Mean Matching (PMM) method generating 20 imputed datasets (*m* = 20). To ensure the accuracy of the results, the imputation model incorporated all variables utilized in the analysis. Subsequent statistical analyses were conducted independently on each imputed dataset, and finally results were combined according to Rubin’s rules. Sensitivity and stability analysis demonstrated the efficiency of the imputation strategy, showing that the findings were robust and reliable. The results are shown in **Supplementary Figure 1**.

### 2.3 Measures

The quantitative assessment measures employed in this study included the YaleBrown Obsessive Compulsive Scale (Y-BOCS), the Self-Rating Anxiety Scale (SAS), the Self-Rating Depression Scale (SDS), and the Symptom Checklist-90 (SCL-90). These instruments are recommended by the DSM-5 and characterized by its simple structure and high applicability and have demonstrated strong reliability and validity in previous research (13, 14).

#### 2.3.1 Y-BOCS

The Y-BOCS is a globally acknowledged instrument for assessing the severity of OCD symptoms. This scale is a semi-structured assessment instrument consisting of 10 items, which must be conducted by trained professionals. Items 1–5 evaluate obsessions (such as intrusive thoughts or images), whereas items 6–10 evaluate compulsions (such as repetitive behaviors or rituals). Each item is scored from 0 points (no symptoms) to 4 points (extreme severity) with a total score of 0 to 40. According to the overall score, symptom severity is classified into four categories: mild or subclinical (0-15), moderate (16-22), severe (23-31), and extreme (32-40). The Chinese version of the Y-BOCS has been widely utilized in previous studies, especially within psychological research aimed at evaluating the effectiveness of clinical interventions (15, 16).

#### 2.3.2 Anxiety and depression

The SAS and SDS are commonly used and reliable tools for self-assessment, frequently employed in psychiatric clinics and psychological centers (17). The scale consists of 20 items. Participants are required to rate the frequency of their own symptoms over the past week on a 4-point Likert scale. Item scores are summed to produce a raw score, which is multiplied by 1.25 to convert the standard score. Anxiety and Depression severity is classified into the following levels: normal (<50), mild to moderate (50-59), moderate to severe (60-69) and severe (≥ 70) (18). To prevent the reduction in statistical efficacy associated with an excessive number of nodes in the network and to minimize the topological overlap with obsessivecompulsive or somatic symptoms, we selected the five most representative items based on previous literature and clinical relevance (12). These items focused particularly on emotional and somatic dimensions (such as psychomotor excitement, crying).

#### 2.3.3 SCL-90

Individuals aged 13 and above can be evaluated by using the SCL-90, which is a widespread self-administered inventory consisting of 90 items. This scale includes nine core symptom domains along with seven supplementary items, focusing frequently on reported symptoms such as appetite disturbances and sleep disorders; but they are not scored as separate dimensions (19). Each symptom subscale contains a cluster of 6 to 13 questions. Participants are required to rate these items based on the 5point Likert scale, where 0 indicates ‘Not at all’ and 4 represents ‘Extremely’. The Global Severity Index (GSI) is calculated as the mean score across all items and serves as an indicator of a person’s overall psychological distress level. It is worth clarifying how “brain function” is operationalized in this study. Rather than relying on neuroimaging data or formal neuropsychological testing, we used Item 90 of the SCL90 (“feeling that something is wrong with your brain”), which taps into patients’ subjective sense that their cognitive processes are malfunctioning. We treat this item as a proxy for perceived cognitive dysregulation: a self-referential appraisal that shares conceptual ground with metacognitive monitoring without directly measuring metacognitive beliefs per se. Accordingly, any mechanistic interpretation involving SCL-90 Item 90 should be read as reflecting patients’ phenomenological experience of mental dysfunction rather than an objective index of brain integrity.

To ensure comparability across instruments, it should be noted that all four measures employed in this study utilize a consistent temporal reference window of “the past week”. Specifically, participants were instructed to rate their symptoms based on their experiences over the seven days preceding the assessment. This uniformity in the time frame mitigates potential confounding that could arise from discrepant recall periods across scales.

### 2.4 Network analysis

#### 2.4.1 Network estimation and visualization

In this study, a Gaussian Graphical Model (GGM) was constructed using the *qgraph* package in R to analyze the network structure among items from the Y-BOCS, SAS, SDS and SCL-90 scales. Given the ordinal nature of the Likert-type item responses, the zero-order correlation matrix serving as input for network estimation was computed using the *cor_auto* function. This procedure automatically detects variable distributions and applies *polychoric* correlations for pairs of ordinal items to account for their discrete measurement properties, thereby mitigating potential bias in parameter estimates. Edges in the graph correspond to regularized partial correlation coefficients after controlling the influence of all remaining nodes (20). This means that the association between any two nodes reflects only their direct relationship after statistically controlling for the potential influence of every other variable included in the model, which is an approach conceptually equivalent to a multivariate regression framework. Consequently, spurious associations driven by shared third variable confounds are effectively eliminated, and only genuine direct connections are retained in the network structure. The core of this approach lies in introducing the LASSO penalty term, which shrinks weak partial correlation coefficients to zero, thus effectively removing spurious links and identifying reliable connections. Due to the limitations in sample size, the hyperparameter (γ) of Extended Bayesian Information Criterion (EBIC) was set to 0.5 in this study. This configuration aims to achieve a balance between ensuring network sparsity and maintaining sufficient sensitivity to identify significant connections. In network visualization, edge colors represent the direction of correlation (green for positive, red for negative), while the thickness of edges corresponds to the strength of the correlation. To evaluate the possible impact of gender factors, we established gender-stratified subnetworks using the same parameter settings. The detailed results can be found in the **Supplementary Material**.

#### 2.4.2 Expected influence and predictability

We employed the *qgraph* package in R to calculate the Expected Influence (EI) of nodes indicating their centrality and significance as critical hubs in the network (21). Furthermore, the *networktools* package was applied to compute Bridge Expected Influence (BEI), quantifying the strength of connections across different symptom communities. To objectively pinpoint key bridge nodes, we adopted a data-driven approach for threshold selection. Specifically, through 1000 bootstrap resampling iterations, the BEI value distribution for each node was established. Nodes with mean BEI values exceed the upper limit of the overall sample distribution were classified as having significant bridge characteristics (22). Concurrently, we assessed nodal predictability by the *mgm* package, reflecting the proportion of a node’s variance that can be explained by its neighboring nodes within the network (23). Predictability reflects how strongly a node is governed by the network structure. Highly predictable nodes can be more effectively influenced indirectly through changes to their neighboring symptoms, whereas items with low predictability are likely affected by external factors outside the network and often require targeted direct intervention. One important point about the network structure needs to be clarified upfront. The network in this study spans five symptom communities: SCL-derived paranoia, SCL-derived psychoticism, depressive symptoms from the SDS, anxiety symptoms from the SAS, and obsessive-compulsive symptoms from the Y-BOCS. This is a more complex setup than a simple two-category model. Because of this, when a node shows a high Bridge Expected Influence value, it does not mean that node only connects two specific symptom categories. Instead, BEI values reflect how much a node transmits information across any combination of these communities. All bridge centrality findings in this paper should be read with this multi-community structure in mind.

#### 2.4.3 Network accuracy and stability

In this study, we assessed the stability and accuracy of the network model using the *bootnet* in R (24). A 95% confidence interval (CIs) for edge weights was constructed via non-parametric bootstrap method (n = 1000) to test the accuracy of the network connections. Narrower CIs suggest accurate estimates of the edge weights. We further employed the case-dropping bootstrap method (n = 1000) to evaluate the stability of EI and BEI. The stability index is based on the Correlation Stability coefficient (CScoefficient). It was defined as the largest proportion of samples that can be excluded from the analysis while still maintaining a correlation above 0.7 between the original centrality measures and those obtained after removal. The stability of Bridge Expected Influence (BEI) was also assessed using the case-dropping bootstrap method (n = 1000). The CS-coefficient for BEI was calculated to determine the reliability of bridge centrality rankings. Following the recommendations of Epskamp et al., the optimal CScoefficient is greater than 0.5, and the minimum acceptable threshold is 0.25. lastly, based on 1000 times bootstrap difference test (α = 0.05), we identified whether there statistically differences among various edge weights and node centrality indicators.

#### 2.4.4 Node redundancy analysis

To evaluate potential item collinearity that could artifactually influence network topology, node redundancy was assessed using the goldbricker function from the networktools package in R (25). This procedure identifies potentially redundant node pairs by comparing the proportion of significantly different correlations each pair exhibits with all other nodes in the network, employing the method of Hittner and colleagues for comparing dependent correlations. Following established conventions, node pairs demonstrating less than 25% significantly different correlations (threshold = 0.25) and a minimum zero-order correlation of 0.5 were flagged as potentially redundant (25). This threshold balances sensitivity to detect meaningful collinearity against the risk of over-identifying spurious redundancies in moderately sized psychological networks.

## 3 Results

### 3.1 The characteristics, mean scores, SD, and predictability for each symptom

The final sample comprised 528 participants with OCD, consisting of 246 females and 282 males. Their ages ranged from 14 to 71 years, with an average of 25.12 ± 9.66 years. **Table 1** provided a detailed list of demographic characteristics along with the mean scores, standard deviations (SD) and predictability for each item of Y-BOCS, SAS, SDS and SCL-90.

**Table 1:** Characteristics, mean scores, SD, and predictability for each symptom of the YBOCS, SCL-90, SAS, and SDS.

Variable	Mean (SD)	Predictability
Gender		
1: Male	282 (53.4%)	
2: Female	246 (46.6%)	
Age (years)	25.12 (9.66)	
SCL7: Mind controlled	0.88 (1.19)	0.98
SCL8: Blamed others	1.44 (1.2)	0.72
SCL16: Auditory hallucinations	0.47 (0.89)	0.85
SCL18: Distrusted others	1.47 (1.37)	1.07
SCL35: Thought broadcasting	0.82 (1.06)	0.69
SCL43: Felt watched	0.95 (1.24)	1.14
SCL62: Alien thoughts	1.39 (1.36)	0.98
SCL68: Unusual thoughts	1.78 (1.4)	1.11
SCL76: Underappreciated	1.06 (1.13)	0.95
SCL77: Lonely in crowd	1.14 (1.25)	1.27
SCL83: Others exploit me	0.64 (1.03)	1.04
SCL84: Distressing sexual thoughts	0.89 (1.24)	0.64
SCL85: Deserve punishment	1.24 (1.29)	0.88
SCL87: Somatic concern	1.41 (1.32)	1.05
SCL88: Lack of closeness	1.27 (1.33)	0.88
SCL90: Brain not right	1.64 (1.39)	1.17
SDS3: Crying spells	1.9 (0.85)	0.89
SDS8: Constipation	1.64 (0.8)	0.40
SDS9: Tachycardia	1.77 (0.83)	0.76
SDS10: Fatigue	2.39 (0.98)	1.14
SDS13: Psychomotor agitation	2.21 (0.95)	0.77
SAS1: Nervous	2.43 (0.92)	1.07
SAS3: Panic	2.47 (0.87)	1.10
SAS4: Mental disintegration	1.94 (0.87)	0.92
SAS8: Easy fatiguability and weakness	2.47 (0.9)	1.02
SAS20: Nightmares	1.7 (0.73)	0.53
YBOCS1: Time and frequency of OC thinking	2.3 (1.07)	0.99
YBOCS2: Social contact or work affected by OC thinking	1.88 (0.97)	0.93
YBOCS3: Distress caused by OC thinking	2.28 (0.97)	0.94
YBOCS4: Resistance to OC thinking	1.73 (0.97)	0.70
YBOCS5: Control of OC thinking	2.31 (0.93)	0.90
YBOCS6: Time and frequency of OC behavior	2.03 (1.07)	0.88
YBOCS7: Social contact or work affected by OC behavior	1.72 (0.99)	1.19
YBOCS8: Distress caused by OC behavior	2.04 (1.09)	1.02
YBOCS9: Resistance to OC behavior	1.66 (0.99)	0.89
YBOCS10: Control of OC behavior	2.1 (1.01)	1.04

### 3.2 Network structure of Y-BOCS, SDS, SAS and SCL-90 in individuals with OCD

**Figure 1:**
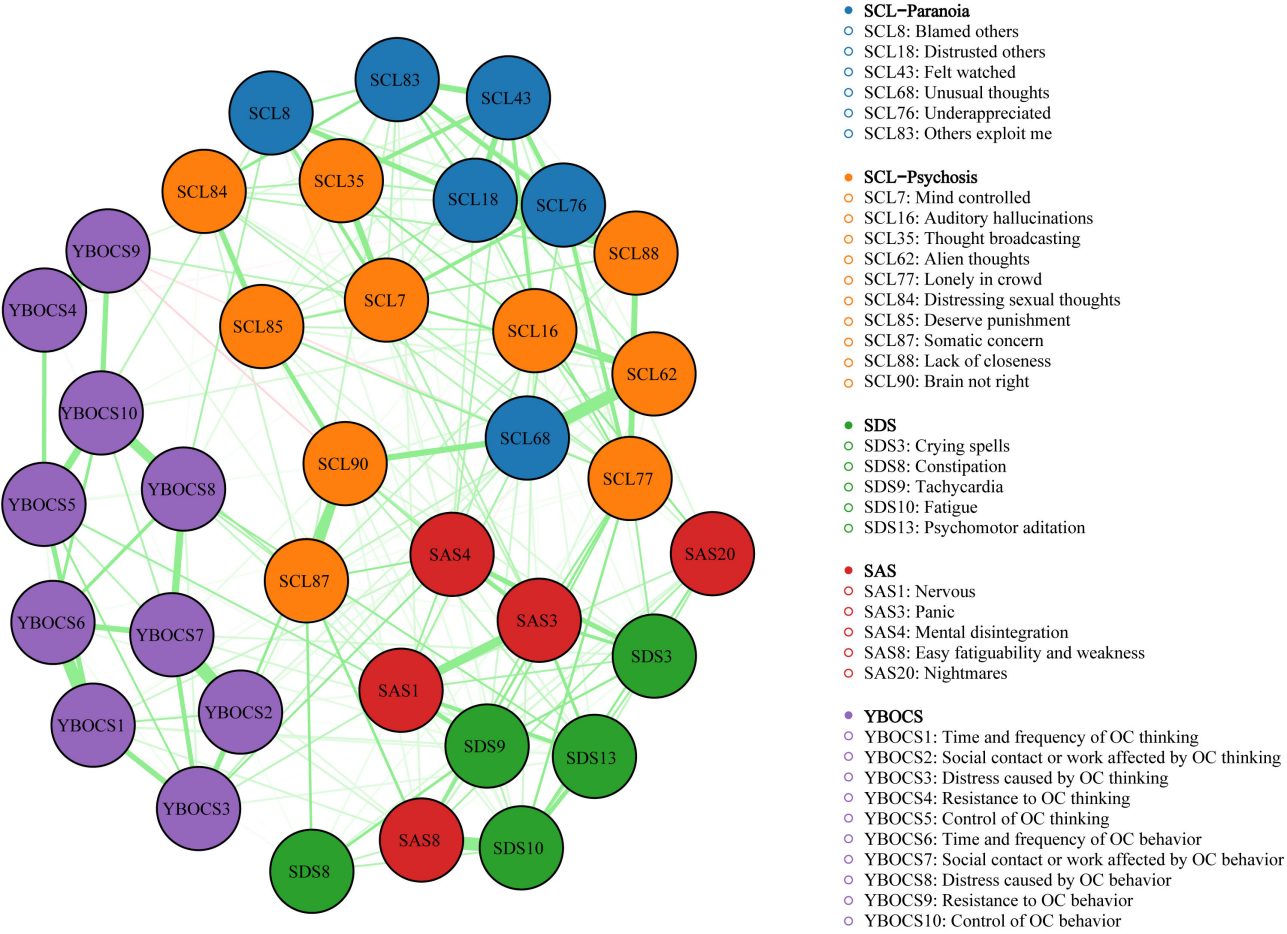
Network structure of mental health symptoms. Green represents positive partial correlations; pink represents negative partial correlations. Edge thickness corresponds to correlation strength. Node predictability is indicated by the surrounding circles. Nodes are color-coded by symptom domain: red (SAS), blue (Paranoia), orange (Psychoticism), green (SDS) and purple (YBOCS).

As illustrated in **Figure 1**, the network model shows complex connectivity patterns. Strong statistical dependencies linking items are found in the same subscales as well as bridging items from different scales. The network consists of 239 edges, with edge weights ranging from -0.05 to 0.53. A total of 127 edges across the scales were identified. It included 29 connections between SCL-90/SAS, 27 between SCL-90/SDS, 29 between SCL-90/Y-BOCS, 18 between SDS/SAS, 15 between SDS/Y-BOCS, 9 between SAS/Y-BOCS. Within the Y-BOCS network, several items displayed notable links to nodes in other symptom clusters. For instance, YBOCS1 exhibited positive correlations with SDS9 and SDS13 in the depression community, SAS1, SAS4 and SAS8 in the anxiety network, as well as SCL62 and SCL87 in the SCL-90 community. The highest connectivity values were recorded for SAS4 (0.04), followed by SAS8 and SDS9 (both 0.03). Conversely, a negative association was detected between YBOCS1 and SDS8.

YBOCS2 was positively correlated with SDS13, SAS8, SCL62, SCL76 AND SCL90, with strongest associations observed for SAS8 and SCL90, showing edge weights of 0.03 and 0.1 respectively.

For the YBOCS3 node, positive links extended to SDS3, SDS13, SAS1, SAS4, SCL68, SCL87 and SCL90. It displayed particularly pronounced connection strengths with SAS1 (*w* = 0.05), SAS4 (*w* = 0.09), SCL68 (*w* = 0.05) and SCL87 (*w* = 0.04).

Only weakly positive ties were identified between YBOCS4 and two items (SDS8, SCL83), whereas a minor negative tie existed with SCL76.

Positive links were observed between YBOCS5 and SDS9, SAS1, SCL8 and SCL90. The edge weights for the connections with SAS1 and SCL8 were 0.08 and 0.06 respectively.

YBOCS6 was positively linked to SDS9 and SCL77 but showed a slight inverse relationship with SDS8.

YBOCS7 showed positive correlations with SAS8, SDS3, SDS13, SCL18, SCL84 and SCL90, demonstrating correlation coefficients of 0.03 for both SDS3 and SDS13.

For the YBOCS8, it exhibited positive associations with SDS13, SAS1, SCL8, SCL76, SCL83, SCL85 and SCL87. Among these, the dominant inks were formed with SCL8 and SCL87, showing weights of 0.07 and 0.06.

Positive associations were identified between YBOCS9 and SDS10, SCL7 and SCL43, and the maximum connection strength with SCL7 was 0.05. In contrast, negative correlations were observed with SCL68 (*w* = −0.03) and SCL90 (*w* = −0.05).

Positive ties connecting YBOCS10 to SDS10, SCL16, SCL85 and SCL87 were observed. In particular, the weight associated with SCL87 stood at 0.07.

Internal connection strengths varied across symptom communities. Within the YBOCS network, we identified 23 edges (weights: 0.01-0.53), with the most robust link occurring between YBOCS4 and YBOCS9. In the SDS network, 9 edges were present (*w*: 0.005-0.11), with SDS8 and SDS9 being most strongly related. The SAS network revealed 6 edges (*w*: 0.06-0.29), led by the SAS1/SAS3 connection. The SCL-90 Community encompassed 74 edges (*w*: 0002-0.39), where SCL62 and SCL68 showed the maximum association.

**Figure 2:**
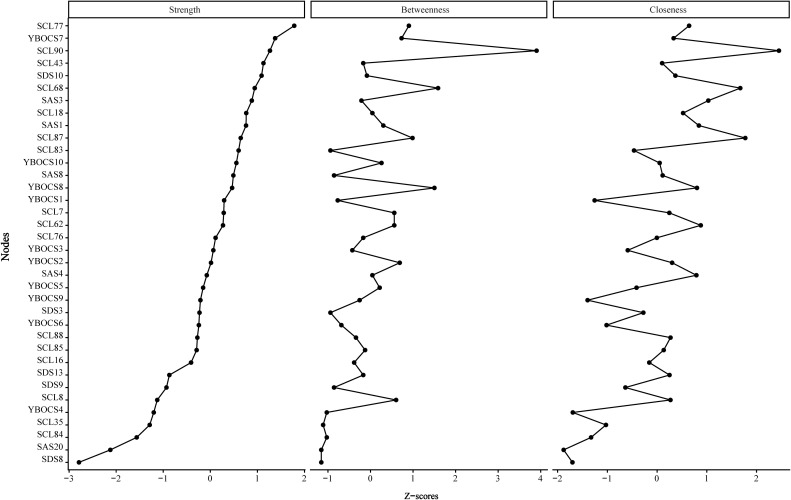
Network centrality profiles individual symptoms. Z-scores for three key centrality measures (Node Strength, Betweenness Centrality, and Closeness Centrality) are visualized for each symptom node. Higher values indicate nodes that are more connected (Strength), act as bridges (Betweenness), or are centrally located (Closeness) in the network.

Node centrality indicators are illustrated in **Figure 2**, including strength, betweenness and closeness centrality. All indicators were standardized by *Z-score* and then ranked. The findings show that SCL90 achieves the highest scores in both betweenness centrality (*w* = 3.90) and closeness centrality (*w* = 2.45), highlighting its pivotal role as a central hub in the network structure. High values in closeness centrality were also observed for SCL87 (*w* = 1.77) and SCL68 (*w* = 1.67). Regarding strength centrality, SCL77 (*w* = 1.78) held the primary position, while YBOCS7 (*w* = 1.38) and SCL90 (*w* = 1.27) ranked second and third, respectively. For betweenness centrality, SCL68 (*w* = 1.59) and YBOCS8 (*w* = 1.50) followed in second and third place. In contrast, SAS20, SDS8 and YBOCS4 exhibited low value across all centrality measures, pointing to their marginal placement in the network.

**Figure 3:**
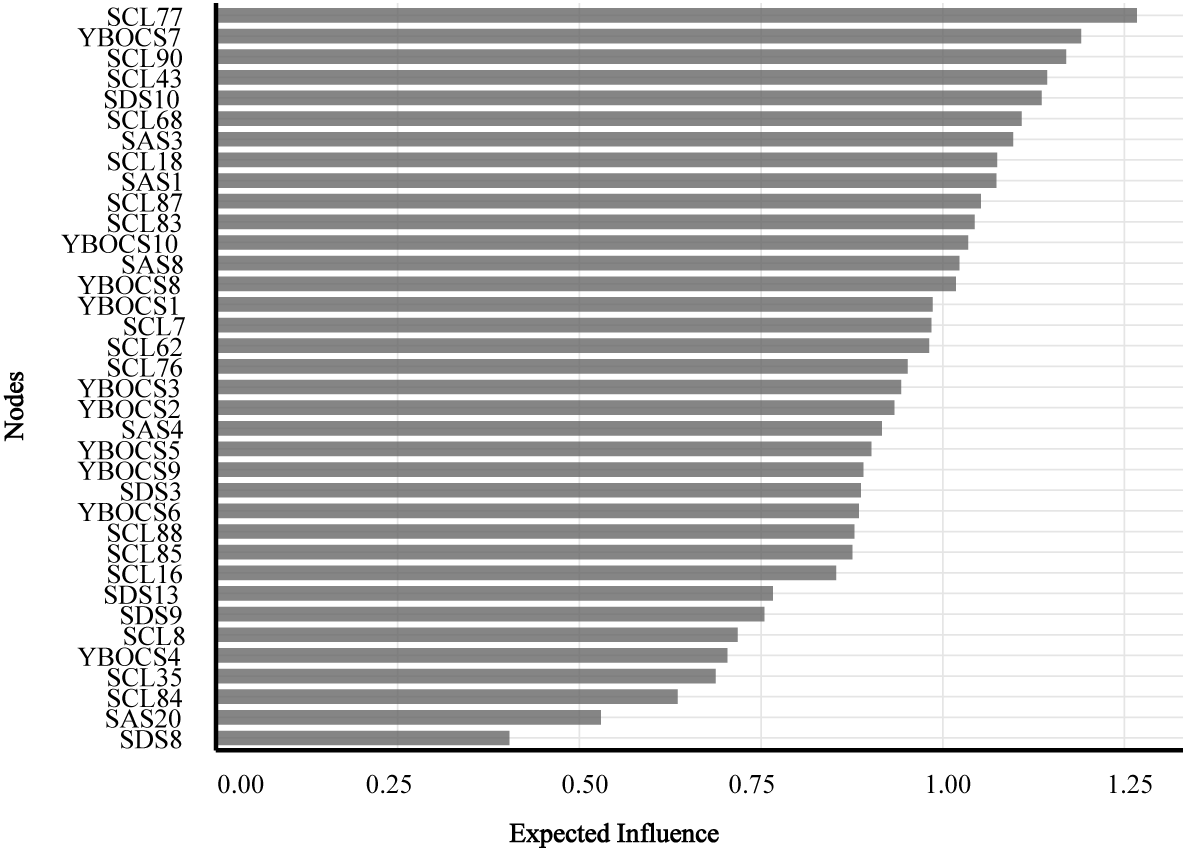
Bar plot shows the expected influence ranking of all nodes in the network. Nodes are arranged in descending order of expected influence values (ranging from 0.00 to 1.25), which represent the cumulative strength of a node’s outgoing connections on the network. Higher values indicate nodes with greater potential to influence other nodes within the network structure.

**Figure 3** depicts the expected influence of the nodes. SCL77, YBOCS7, SCL90, SCL68 and SCL87 displayed significantly higher expected influence values, suggesting their central role or strong connectivity in the network structure. Conversely, SDS8 recorded the minimum expected influence. Notably, EI values were all positive, consistent with the predominance of positive partial correlations in the estimated network.

**Figure 4:**
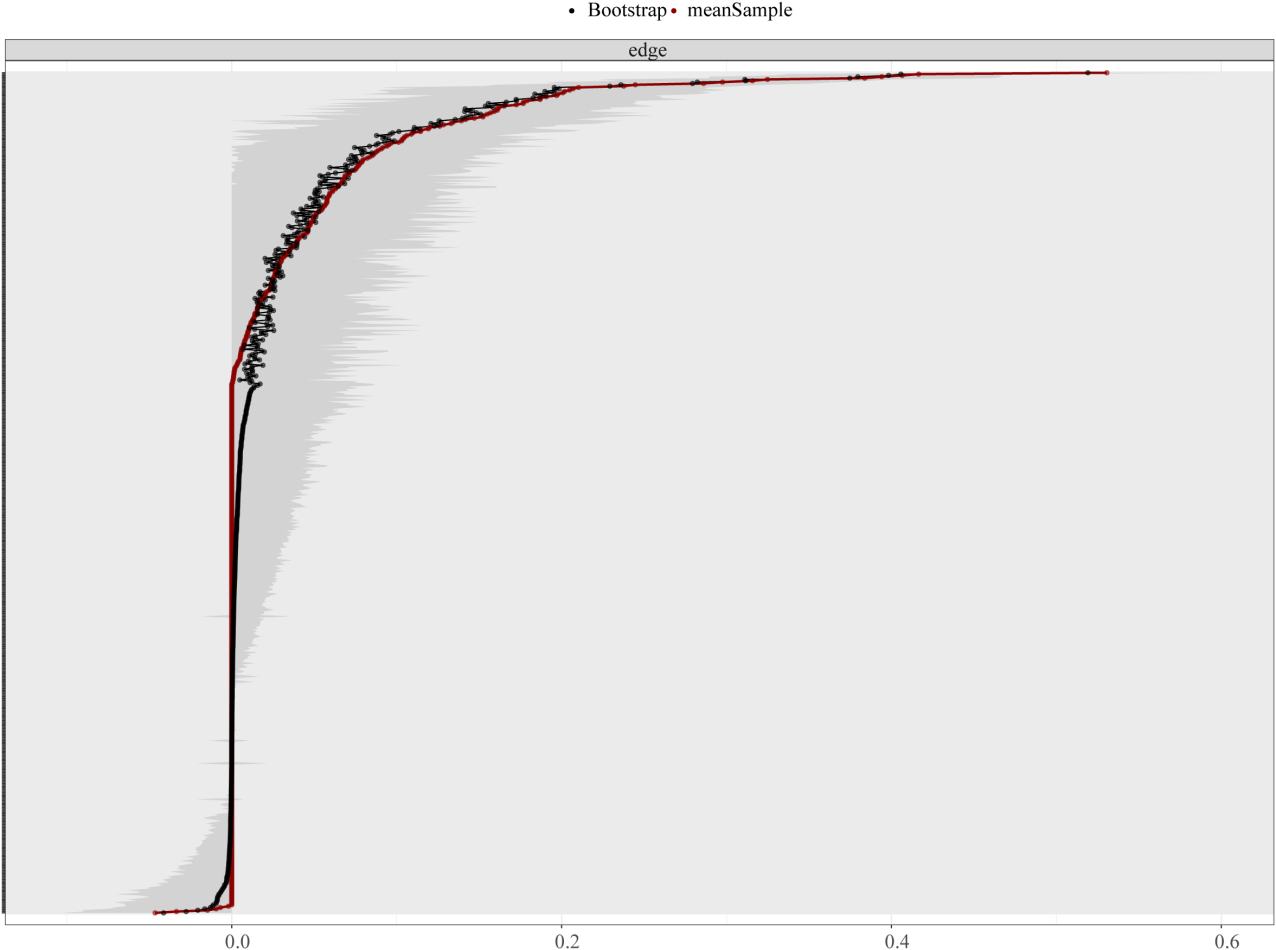
Edge weight accuracy evaluation. Comparison between original sample edge weights and bootstrap means across the observed range of edge weights (0.0-0.6). Close agreement between these measures supports the reliability of the network structure.

The robustness of the network was evaluated by using the Bootstrap method. As shown in **Figure 4**, the 95% confidence interval for edge weights is quite narrow, implying high estimation accuracy. The dominant associations across distinct symptom communities were observed in the pairs of YBOCS2/SCL90 (*w* = 0.11), YBOCS3/SAS4 (*w* = 0.09) and YBOCS8/SDS13 (*w* = 0.08).

**Figure 5:**
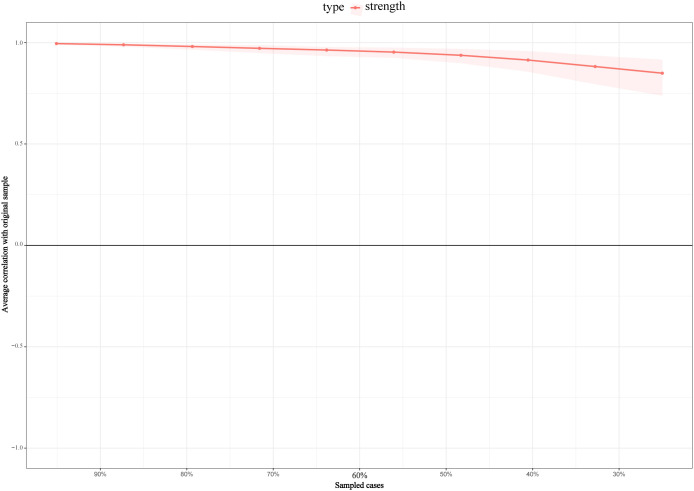
Network stability analysis. The average correlation (y-axis) between the centrality order of the original sample and the order estimated from bootstrap subsamples is plotted against the percentage of cases retained (x-axis). The analysis progressively drops cases from 90% to 30% of the original sample size. The results demonstrate the robustness of the centrality rankings to variations in the sample.

Network stability was assessed via the Case-dropping bootstrap method (**Figure 5**). The average CS-stability coefficient of the three centrality indices was 0.47, substantially exceeding the critical lower limit of 0.25, which confirmed the network’s reliability. The CS-coefficient for BEI was 0.67, exceeding the recommended threshold of 0.5, suggesting acceptable stability of the bridge centrality estimates. Results revealed that the network structure maintained a strong correlation with the original network even when the sample size was decreased to 50%. Even though reducing the sample size further to 30% it had little impact on the coefficient correlation, suggesting that the current network model possessed excellent robustness and reproducibility.

### 3.3 Demographic correlates of network measures

Gender-stratified descriptive statistics are presented in **Table 2** (**Supplementary Materials**). Male participants (n = 282) had a mean total symptom score of 57.03 (SD = 22.54), slightly lower than females (n = 246; mean = 60.70, SD = 23.89). Core symptom severity, defined as the average score across the four central network nodes (SCL90, SCL77, SCL68, SCL87), was 1.45 (SD = 1.04) for males and 1.54 (SD = 1.11) for females. The individual network activation index, which weights symptom load by node strength centrality, yielded means of -1.61 (SD = 20.15) and 1.85 (SD = 21.24) for males and females respectively. Welch’s independent samples t-tests found no statistically significant gender differences for total symptom severity (t [506.78] = 1.81, p = .071), core symptom severity (t [505.10] = -1.03, p = .305), or network activation scores (t [507.82] = -1.92, p = .056), though the latter two comparisons approached marginal levels.

**Table 2:** Demographic comparisons and age-related associations of symptom measures.

Measure	Males (n = 282)	Females (n = 246)	Gender difference	Age correlation
	M (SD)	M (SD)	*p*	*r*
Total symptom severity	57.03 (22.54)	60.70 (23.89)	.071	-.097
Core symptom severity^a^	1.45 (1.04)	1.54 (1.11)	.305	-.080
Network activation score^b^	-1.61 (20.15)	1.85 (21.24)	.056	-.095

Gender differences (Welch's *t*-test): Total symptoms, *t*(506.78) = -1.81, *p* = .071; Core symptoms, *t*(505.10) = -1.03, *p* = .305; Network activation, *t*(507.82) = -1.92, *p* = .056. Age correlations (Pearson's *r*, df = 526): Total symptoms, *r* = -.097, *p* = .026; Core symptoms, *r* = -.080, *p* = .067; Network activation, *r* = -.095, *p* = .029. Network activation robustness: Spearman ρ = -.104, *p* = .017; Partial *r* (controlling for gender) = -.099, *p* = .022. ^a^Core symptom severity = mean of SCL90, SCL77, SCL68, and SCL87. ^b^Network activation score = individual weighted symptom burden based on node strength centrality from the 36-node network.

We also examined how age related to network-derived outcomes using Pearson correlations. Age showed a small but significant negative correlation with total symptom burden (r [526] = -.097, p = .026) and with individual network activation scores (r [526] = -.095, p = .029), suggesting that younger patients tended to show higher alignment with the central network nodes. The correlation between age and core symptom severity did not reach significance (r [526] = -.080, p = .067). A supplementary Spearman’s rank correlation produced consistent results (ρ = -.104, p = .017), supporting the finding that network involvement tends to be higher among younger patients.

To explore potential gender differences in the symptom network of OCD disorder, distinct networks were built for male and female participants. The results are shown in **Supplementary Figures 2** and **S3**. A systematic comparison was conducted focusing on global network metrics, specific edge weights and node centrality. Our analysis of global network characteristics demonstrated that total network strength did not differ significantly across genders (*w* = 0.740). Nevertheless, the network structure invariance test indicated a borderline difference in global network (*w* = 0.098), suggesting that the association patterns among symptoms might be varied by gender.

### 3.4 Node redundancy analysis

The Goldbricker analysis encompassed all 36 nodes and systematically evaluated a total of 630 unique node pairs. None of the pairs satisfied the predefined redundancy criterion: every one of the 630 pairs produced suggested reduction values that remained above the threshold of 0.25, and no pair attained statistical significance at p = .05. To further characterize the degree of inter-node similarity, the 24 node pairs exhibiting the lowest suggested reduction values were examined as those showing the closest conditional association patterns. The highest degree of similarity was observed between SCL76 and SCL18, which returned a suggested reduction value of 0.088. The next most similar pairs were YBOCS3 and YBOCS2, and YBOCS7 and YBOCS2 (suggested reduction = 0.118). Nonetheless, not one of these pairs surpassed the redundancy threshold, providing evidence that every node contributed non-overlapping variance to the overall network structure.

## 4 Discussion

This research represents the initial application of network analysis to a sample of 528 outpatients with OCD, constructing an integrated psychopathological network that includes obsessive-compulsive symptoms, psychotic and paranoid traits and the emotions of anxiety and depression. Results indicated that SCL90 serves as a primary connector evidenced by tis dominance in betweenness and closeness centrality, positioning the subjective cognitive dysfunction as the key hub linking OCD pathology with other constituent symptom domains within the multi-community framework. Given the cross-sectional nature of the data, however, the directional flow of these associations remains indeterminate; SCL90 may function as either a conduit through which distress propagates or a point of convergence where multiple symptom pathways intersect. In terms of local connectivity, the peak node strength of SCL77 points to perceived social isolation as the most substantial psychological burden for these patients. Additionally, the network structure revealed distinct inter-symptom bridges that facilitate connectivity across the constituent symptom communities. For instance, edges connecting obsessive distress to SDS13 and obsessive interference to SCL90 exemplify cross-domain pathways through which activation may propagate between the obsessive-compulsive, depressive, and metacognitive dimensions of the network. Beyond the identification of individual hub nodes, the pattern of cross-domain edges warrants specific attention. Across the 127 inter-community connections identified in the network, several notably strong edges emerged. The most prominent cross-domain associations were observed between YBOCS2 and SCL90 (*w* = 0.11), between YBOCS3 and SAS4 (*w* = 0.09)), and between YBOCS8 and SDS13 (*w* = 0.08)). Notably, the single strongest cross-domain edge overall was between SDS10 and SAS8 (*w* = 0.41)), underscoring the substantial symptomatic overlap between depressive and anxious symptomatology. Additionally, a dense cluster of moderate cross-domain connections linked SCL-90 psychoticism items (particularly SCL68 and SCL87) to both Y-BOCS and SAS nodes, reinforcing the structural integration of psychotic-like experiences within the broader OCD symptom network.

It warrants reiteration that the network under investigation was specified across multiple symptom communities rather than a binary diagnostic framework. Consequently, references to bridging or cross-domain connectivity throughout this discussion should be understood as denoting transmission across any combination of the constituent communities, consistent with the analytic assumptions detailed in the Methods. The most striking finding of this study reveals that SCL90 exhibited the highest values in terms of betweenness and closeness centrality. Given the crosssectional design, SCL90 may serve as a potential mediator, though causal directionality cannot be established. Previous investigations employing the Y-BOCS have demonstrated that poor insight is closely related to high symptom severity (8, 26–28). Research indicates that around 36% of individuals with OCD exhibit poor insight or delusional beliefs. This subgroup typically suffers from more severe OCD symptoms, depressive states and functional impairments (29). Additional study suggests that people with alexithymia struggle to identify and express their emotional states, often displaying more intense symptomatology, a stronger sense of personal responsibility and an elevated risk of suicidal thoughts (30). Clinical evidence consistently validates that severe OCD is strongly associated with substantial impairments in occupational functioning, family dynamics and social engagement (31). Beyond mere somatic complaints, the high betweenness centrality of SCL90 suggests that the subjective perception of cognitive dysfunction may play a bridging role in the symptom network that is consistent with metacognitive processes. However, given that this item operationalizes a self-reported sense of perceived cognitive disturbance rather than a direct measure of metacognitive beliefs or objective brain function, its theoretical implications should be interpreted as a hypothesized mechanism requiring longitudinal verification, rather than an established causal account. Specifically, this beliefs as the patient’s conviction that an inability to control thought content signifies a catastrophic breakdown of their neurological functioning. In the context of OCD, patients are persistently troubled by ego-dystonic intrusive thoughts and are compelled to perform exhausting compulsive behaviors. This pattern closely aligns with Well’s metacognitive model, which posits that metacognitive beliefs have a greater influence on the emergence of symptoms than the specific nature of the cognitive content itself (26). According to the Self-Regulatory Executive Function (S-REF) theory, the tendency to interpret obsessive thoughts negatively arises from the activation of specific metacognitive beliefs, especially those regarding thought-fusion (27). Research indicates that metacognition serves not only as a transdiagnostic vulnerability factor for various psychological disorders (28). but also exhibits a strong association with specific symptom dimensions in OCD (32). Moritz and colleagues found that the need for thought control and negative beliefs regarding uncontrollability and danger demonstrated the strongest correlation with OCD symptom severity. This profound mistrust of one’s own mind may compromise the patient’s capacity for reality testing, making SCL68 develop into Psychotic-Like Experiences (PLEs) (33). Such findings provide compelling support for the Inference-Based Approach (IBA). The core premise of the IBA model posits that obsessions are not merely intrusive thoughts, but rather obsessional doubts inferred about reality (34, 35). This framework highlights obsessive beliefs and pathological doubt arise from a particular cognitive distortion characterized by intense skepticism toward immediate sensory input, combined with an overdependence on introspective reasoning regarding imagined possibilities (10).

Despite the dominance of SCL90 in controlling network traffic, the highest centrality strength was observed in SCL77 (lonely in crowd; Z = 1.78), which also ranked first in expected influence across the entire network (EI = 1.25). This indicates that SCL77 is not only the most activated node but also the one most intricately connected with other symptom nodes. Loneliness may represent a central maintaining factor, a hypothesis that requires longitudinal verification. One interpretive limitation deserves attention here. The high centrality of SCL-77 points to perceived social isolation as a significant feature of OCD symptom networks, with strong ties to negative social cognitions, emotional disconnection, and related processes. This may help explain the social and affective difficulties commonly seen in OCD patients. However, a single item like SCL-77 cannot cleanly capture “loneliness” alone. It likely also taps into related experiences such as alienation, a sense of not belonging, depressive affect, and social anxiety, making it hard to attribute its centrality to loneliness specifically. Future studies using more precise tools, such as the UCLA Loneliness Scale, combined with network or latent variable approaches, would allow for cleaner separation of these overlapping constructs. It should also be noted that the relatively low centrality of SDSderived nodes does not mean depressive symptoms are clinically unimportant in OCD. As described in the Methods section, only five SDS items were selected, focused mainly on emotional and somatic features (e.g., psychomotor agitation, crying spells), specifically to avoid redundancy with obsessive-compulsive symptom items. This selection constraint, consistent with standard practice in OCD network studies (12), limits how prominently depressive symptoms can emerge as central hubs. The redundancy analysis in Section 3.4 confirmed that SCL77 was not flagged as redundant with any other node, indicating that its high centrality reflects a genuinely distinct structural position rather than an artifact of the restricted SDS representation. The elevated centrality of SCL77 therefore reflects a mechanistically distinct role that perceived isolation plays within OCD pathology. Loneliness carries distinct theoretical significance within the context of obsessive-compulsive disorder, operating through a social-cognitive pathway that is partially independent of, and upstream from, classical depressive symptomatology. The ego-dystonic and socially conspicuous quality of compulsive behavior engenders acute shame, driving systematic withdrawal from social contexts (36, 37). This withdrawal generates a subjective sense of isolation that persists regardless of objective social proximity, a state captured precisely by the notion of being lonely in a crowd. Such perceived isolation attenuates the buffering capacity of social support against symptom exacerbation while simultaneously reinforcing depressive affect through ruminative cognitive processing, thereby establishing a selfperpetuating maintenance cycle. Notably, the subjective experience captured by SCL77, which may encompass loneliness, alienation, thwarted belongingness, and depressive affect, does not appear to merely co-occur with depression at the network level; its strength centrality and expected influence values substantially exceed those of all SDSderived nodes, a pattern consistent with the hypothesis that perceived social isolation may exert an influence on depressive effect through a distinct social-cognitive route. Nevertheless, given both the cross-sectional design of this study and the inherent construct-specificity limitations of a single item, this proposed directional relationship should be regarded as a tentative mechanistic hypothesis requiring prospective empirical validation rather than an established causal account. This mechanistic specificity is supported by extensive empirical evidence linking loneliness independently to a range of mental health conditions, including depression, generalized anxiety disorder, OCD, and PTSD, above and beyond shared variance with general negative effect (38, 39). Existing literature regarding Obsessive-Compulsive and Related Disorders (OCRDs), especially body dysmorphic disorder, have shown that a staggering 96% to 100% of patients suffer from moderate to severe impairments in social functioning (40, 41). Consequently, the centrality of loneliness in the OCD network is underpinned by a unique pathological mechanism and should not be misconstrued as merely occupying the functional niche otherwise filled by depression. We propose that Exposure and Response Prevention (ERP) monotherapy focusing solely on obsessivecompulsive symptoms may achieve constrained outcomes without concurrent attention to the patient’s deep-seated feelings of loneliness.

The Schizo-Obsessive Spectrum hypothesis is substantiated at the network level by the prominent expected influence and closeness centrality identified in SCL68 (unusual thoughts) and SCL87 (somatic concerns). This points to a potential intermediate zone where these nodes occupy structurally strategic positions facilitating transmission from obsessions toward overvalued ideas and delusional states, within the multi-community context. In our network, these nodes are not disconnected but are intricately linked with specific OCD symptoms. This implies that in certain individuals with OCD, the relationship between compulsion and PLEs is not merely co-occurring but share underlying pathological mechanisms. Fenton and McGlashan reported that approximately 12.9% of schizophrenia patients exhibited comorbid obsessivecompulsive symptoms (42); Eisen and Rasmussen observed that roughly 14% of OCD patients displayed psychotic features such as hallucinations or delusions (5); Poyurovsky and partners further revealed that there is no significant correlation between the severity of OCD symptoms and core schizophrenic symptoms in comorbid patients (43). These findings implied that the schizophrenia-obsessive subtype might possess distinct pathophysiological characteristics and profound social dysfunction. Recently investigations have indicated that specific phenotypic characteristics of OCD can predict the transformation of PLEs to clinical psychosis (44). This potential pathological connection may explain why a subset of patients with treatment-resistant OCD show a poor response to Selective Serotonin Reuptake Inhibitors (SSRIs) monotherapy, requiring the addition of antipsychotic drugs for enhanced treatment.

Analysis of edge weights identified possible cyclical pathways of symptom exacerbation. A strong linkage (*w* = 0.08) between YBOCS8 (distress caused by OC behavior) and SDS13 (psychomotor aditation) delineates a specific mechanism of the transformation from OCD to affective disorders. The intense emotional distress arising from unfulfilled compulsions is directly externalized as physical restlessness and agitation, which is regarded as a precursor to elevated suicide risk. Previous studies have confirmed that OCD patients with a high risk of suicide are usually accompanied by more severe obsessive-compulsive symptoms, depressive moods and functional impairments (45). Velloso and colleagues considered suicide risk as a continuous spectrum ranging from no tendency to ideation and finally to attempts. They found that patients at the high-risk end of this spectrum exhibited more severe depressive and obsessive-compulsive symptoms (46). A more pronounced edge (*w* = 0.11) was formed between YBOCS2 (social contact or work affected by OC thinking) and SCL90 (brain not right), constituting potential negative feedback loop. In this cycle, as the interference of obsessive symptoms in daily life becomes more severe, patients become increasingly convinced that their brain is broken. This catastrophic perception of their cognitive abilities reflecting a breakdown in metacognitive beliefs, further undermines their confidence and capacity to resist compulsive behaviors. Combining the observed network structure with existing theoretical accounts, three possible mechanisms may explain how the central nodes contribute to the persistence and worsening of OCD symptoms.

One plausible mechanism involves a metacognitive collapse cycle. When obsessive thoughts begin to disrupt everyday activities (YBOCS2), individuals may develop a catastrophic belief that their mind is fundamentally defective (SCL90; w = 0.11). Wells’ S-REF model offers a relevant framework here: once confidence in one’s own mental control erodes, intrusive thoughts become harder to dismiss, resistance to obsession weakens, and the compulsion to perform rituals intensifies. The more compulsive rituals accumulate, the more daily functioning deteriorates, which in turn deepens the conviction that the brain is broken and sustains a self-reinforcing cycle. This perspective raises the possibility that therapies targeting metacognitive beliefs and insight, such as Metacognitive Therapy or the Inference-Based Approach, may be more effective at interrupting this process than approaches that address surface-level symptoms alone.

A separate but equally important process concerns shame, social withdrawal, and rumination. The socially visible and ego-dystonic nature of compulsive behaviors tends to generate intense feelings of shame, which pushes patients away from social contact. The resulting retreat cultivates a profound sense of subjective isolation, even in the physical presence of others (SCL77), thereby removing the buffering effects that social connection ordinarily provides. Over time, this state of isolation feeds ruminative thinking that deepens depressive mood and sharpens obsessive preoccupation. The distress that accumulates through this process, together with the continuing salience of ego-dystonic symptoms, circles back to reinforce shame and further withdrawal, sustaining a closed maintenance loop. The high strength centrality of SCL77 (Z = 1.78) and its top-ranking expected influence are structurally consistent with this proposed role as an active cross-community hub, rather than a peripheral correlation of illness burden.

A distinct pathway can also be identified linking confused inferential processes to psychotic-like experiences. In line with the Inference-Based Approach, an overreliance on internal doubt rather than sensory evidence, reflected in unusual thought content (SCL68) and heightened bodily preoccupation (SCL87), may facilitate crosscommunity transmission between obsessional and psychotic-like symptom dimensions. The elevated closeness centrality of both SCL68 (Z = 1.67) and SCL87 (Z = 1.77) points to their close structural ties with multiple symptom clusters, positioning them as potential entry points through which obsessive pathology could progress into the schizo-obsessive domain. This interpretation aligns with longitudinal findings indicating that OCD symptoms can predict the transition of psychotic-like experiences into full clinical psychosis (44).

Across all three proposed mechanisms, an important caveat applies. Given the cross-sectional nature of this study, the directional flow described above cannot be confirmed by the present data. The pathways outlined here are best understood as theoretically grounded hypotheses, to be tested in future work using longitudinal designs or experimental methods. In addition to delineating the core network architecture and its mechanistic implications, we examined the extent to which demographic characteristics modulate the network derived indices reported above, an analysis that serves both as a robustness check and as an evaluation of the generalizability of the identified core nodes across demographic strata. With respect to gender, no statistically significant differences emerged for total symptom severity (t [506.78] = -1.81, p = .071), core symptom severity (t [505.1] = -1.03, p = .305), or individual network activation scores (t [507.82] = -1.92, p = .056). The consistent absence of significant effects across these complementary metrics converges with the Network Comparison Test results, which demonstrated invariance in global network strength between male and female participants (p = .740). This structural equivalence indicates that the core network topology generalizes across genders, implying that the network informed intervention targets identified herein, including metacognitive restructuring for subjective cognitive dysfunction and interpersonal approaches for perceived social isolation, are unlikely to require substantive gender-based modification. In contrast to these null-gender findings, chronological age exhibited a modest but statistically reliable negative association with total symptom severity (r = -.097, p = .026) and network activation scores (r = -.095, p = .029), an association that persisted after controlling for gender (r_partial = -.099, p = .022). The negative correlation with core symptom severity did not attain statistical significance (r = -.080, p = .067), suggesting that the age effect may be diffusely distributed across the broader symptom network rather than concentrated exclusively within the principal hub nodes. This age-related attenuation is consistent with developmental models positing that metacognitive capacities continue to mature across adolescence and into early adulthood, particularly the ability to disengage from catastrophic appraisals of cognitive functioning. Older individuals may possess more elaborated strategies for reappraising the significance of intrusive thoughts and for mitigating the shame driven social withdrawal cycle described previously. From a clinical standpoint, this developmental gradient implies that younger patients, who exhibit elevated network activation scores and greater alignment with the high centrality core, may derive disproportionate benefit from early and intensive intervention aimed at dismantling the central symptom axis before maladaptive couplings become fully entrenched.

## 5 Conclusions

This study used network analysis to map the symptom structure of OCD in a sample of 528 outpatients. The key finding was that subjective cognitive dysfunction (SCL90) served as a central connector in the network, linking obsessive-compulsive symptoms to broader psychiatric clusters such as paranoia and somatization. Perceived loneliness (SCL77) showed the highest strength centrality and expected influence overall, pointing to social isolation as an active and potentially self-sustaining component of OCD pathology rather than a mere consequence of illness. Because standard ERP does not directly address social isolation, adding interventions such as Interpersonal Psychotherapy or group-based formats may be worth considering for patients where loneliness appears prominent. For SCL90, approaches targeting metacognitive beliefs, such as Inference-Based Therapy or Metacognitive Therapy, may help patients rebuild confidence in their own thinking. Future treatment models should look beyond symptom reduction and give more attention to restoring patients’ sense of mental control and social connection. Compared with earlier OCD network studies that focused mainly on anxiety-depression comorbidity (12), this study makes three contributions. First, it is the first to include items from both the psychoticism and paranoid ideation subscales of the SCL-90 within a multi-community OCD network, filling a gap in how psychotic-like features have been handled at the item level. Second, the elevated bridge centrality of SCL68 and SCL87, identified through bootstrap-based thresholding, provides specific node-level evidence for how obsessional symptoms may connect to psychotic-like experiences, going beyond simple co-occurrence rates. Third, perceived loneliness (SCL77) emerges here not as a peripheral correlation of OCD burden but as a structurally central node with the highest strength and expected influence in the network, suggesting it plays a distinct mechanistic role that warrants targeted clinical attention. These findings should be interpreted cautiously given the cross-sectional design, and future longitudinal work is needed to confirm the proposed pathways.

## Data Availability

The raw data supporting the conclusions of this article will be made available by the authors, without undue reservation.
