# Supplementary figures and images for: “Brain not right” and “lonely in a crowd”: unveiling the central architecture of psychopathology in obsessive-compulsive disorder
Source: Front Psychiatry. 2026 May 18;17:1823876. doi: 10.3389/fpsyt.2026.1823876 (PMC13222994; doi:10.3389/fpsyt.2026.1823876)

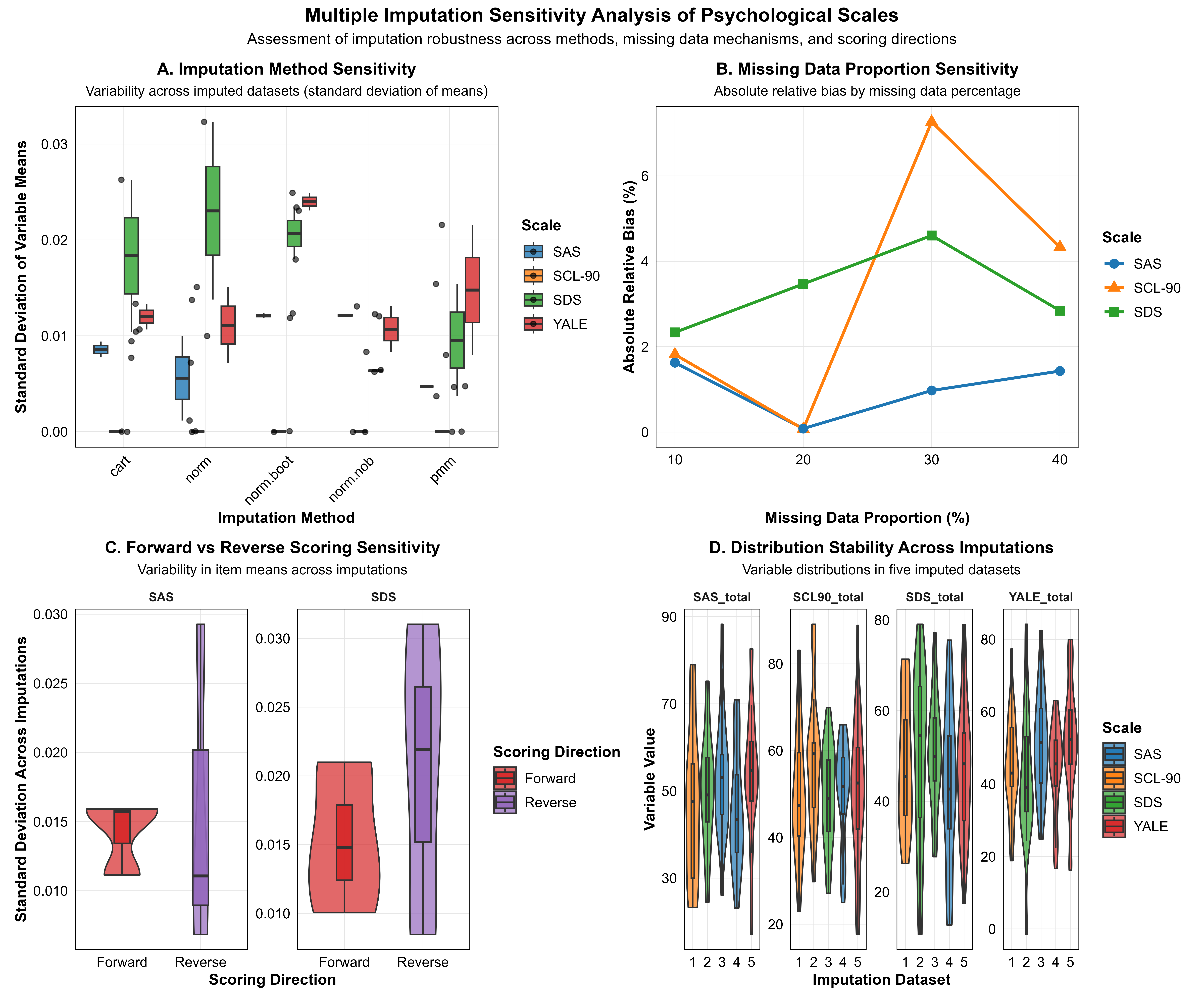

Supplement: Supplementary file 1 [file Image1.tiff]

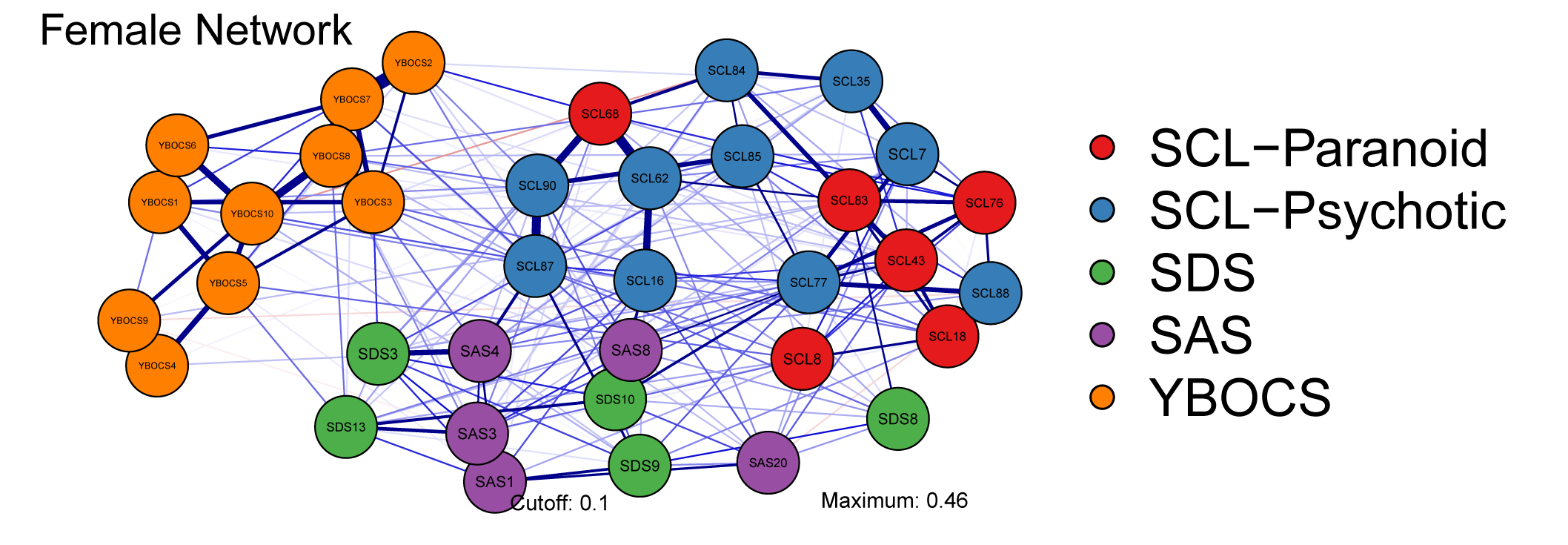

Supplement: Supplementary file 2 [file Image2.tif]

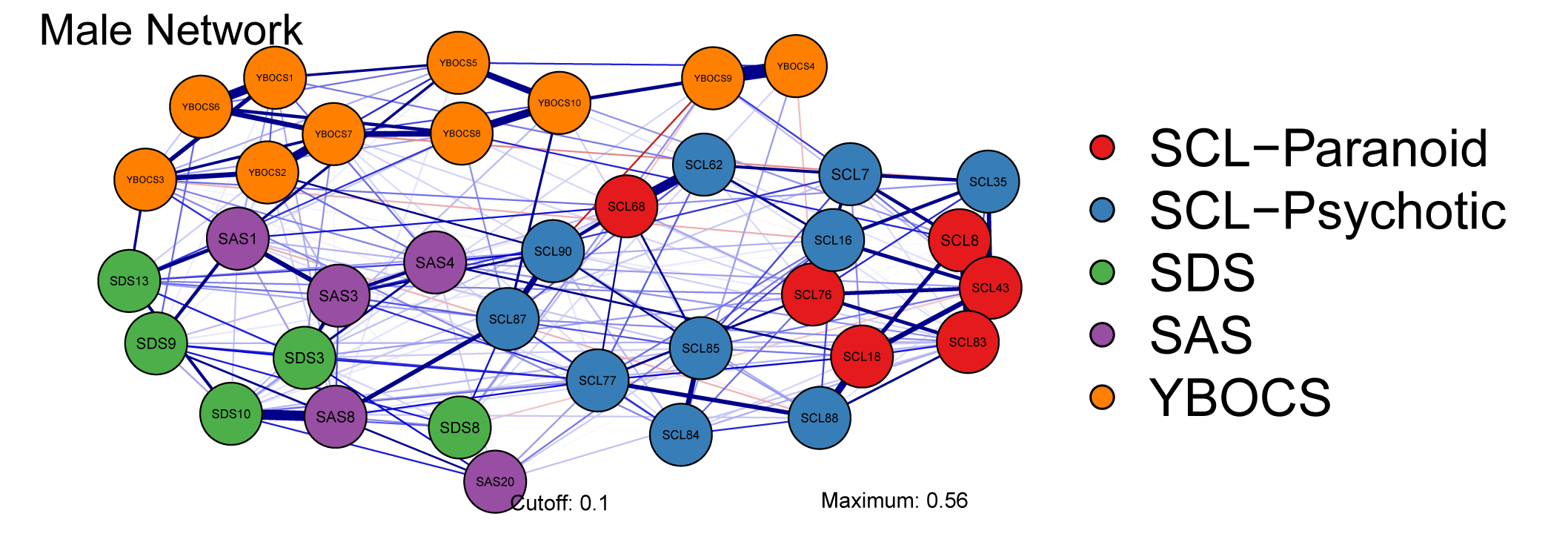

Supplement: Supplementary file 3 [file Image3.tif]
